# 3D-Printed Fast-Dissolving Oral Dosage Forms via Fused Deposition Modeling Based on Sugar Alcohol and Poly(Vinyl Alcohol)—Preparation, Drug Release Studies and In Vivo Oral Absorption

**DOI:** 10.3390/pharmaceutics15020395

**Published:** 2023-01-24

**Authors:** Sorato Ikeda, Masanori Kobayashi, Soken Aoki, Takayuki Terukina, Takanori Kanazawa, Hiroyuki Kojima, Hiromu Kondo

**Affiliations:** 1 Pharmaceutical Research and Technology Labs., Technology, Astellas Pharma Inc., 180 Ozumi, Yaizu, Shizuoka 425-0072, Japan; 2 School of Pharmaceutical Sciences, University of Shizuoka, 52-1 Yada, Suruga-ku, Shizuoka 422-8526, Japan

**Keywords:** fused deposition modeling, fast dissolving, filament formulation, printed medicine, sugar alcohol, PVA

## Abstract

Three-dimensional printing technology holds marked promise for the pharmaceutical industry and is now under intense investigation. Most research is aimed at a greater efficiency in printing oral dosage forms using powder bed printing or fused deposition modeling (FDM). Oral dosage forms printed by FDM tend to be hard objects, which reduce the risk of cracking and chipping. However, one challenge in printing oral dosage forms via FDM is achieving rapid drug release, because the materials for FDM are basically thermoplastic polymers with slow drug release properties. In this study, we investigated printing a fast-dissolving oral dosage form by adding sugar alcohol to a poly(vinyl alcohol)-based formulation for FDM. Filaments which contain sugar alcohol were successfully prepared, and objects were printed with them as oral dosage forms by FDM. On drug release testing, a printed oral dosage form in a ring shape which contained 55% maltitol showed a more than 85% drug release in 15 min. In vivo oral absorption of this printed oral dosage form in dogs was comparable to that of a conventional fast-dissolving tablet. Of particular interest, the drug release profile and drug amount of the oral dosage forms can be easily controlled by a change in shape using 3D Computer Aided Design. These characteristics will encourage the prevalence of FDM by the pharmaceutical industry, and contribute to the promotion of personalized medicine.

## 1. Introduction

Three-dimensional printing technology is a useful tool for creating objects with a shape freely designed by a 3D Computer Aided Design (CAD) system. This technology first gained the attention of the manufacturing industry thanks to its ease of rapid prototyping and lack of the need to prepare molds.

This technological wave has now reached the healthcare industry. Some medical devices, for example casts and artificial limbs, can be customized with a 3D printer to individual patient needs. For example, 3D-printed artificial trachea and bones have been surgically implanted in patients [1,2,3,4]. In addition to medical devices, the first 3D-printed tablet, Spritam^®^ (levetiracetam), was approved by the FDA in 2015. Spritam^®^ is a fast-dissolving 3D-printed tablet based on ZipDose^®^ technology [5]. Further, Merck and AMCM/EOS announced a cooperation agreement on the development of 3D-printed tablets in 2020, aimed at enabling flexible and sustainable local tablet production according to specific market requirements as well as adaptation to patient needs [6].

A major advantage of 3D printing technology is that it allows the creation of any type of complex shape and geometry in a flexible manner. For oral dosage forms, a change in shape means not only a change in appearance but also in the dose and drug release profile of the dosage form [7]. This technology might therefore aid the promotion of personalized medicine, and research aimed at applying it to the preparation of oral dosage forms is now actively underway [8].

Among the many types of 3D printing now available, the four main methods are stereolithography (SLA), selective laser sintering (SLS), inkjet 3D printing and fused deposition modelling (FDM). All of these can be used to print oral dosage forms [9,10].

In SLA, a liquid resin is solidified layer by layer via polymerization by UV irradiation. Objects are fabricated in a liquid bath filled with liquid resin [11]. For SLS printing, a flattened powder is fused and solidified with a laser beam. After solidifying the powder in the first layer, the second powder layer is added onto the first and the fusing and solidification process is repeated, layer by layer. In the case of printing medicines, drug substance is added in a powder layer [12]. Inkjet 3D printing also creates objects in a powder bed, like SLS. In this method, a liquid binder solution is sprayed onto a flattened powder. Powder particles which contact the binder solution then become adherent to each other. Once the initial layer is printed, a new layer of powder is set on that printed layer and the binding solution is again sprayed on this second layer. Three-dimensional objects are created by repeating this process. In the case of printing medicine, drug substance can be loaded in either the binder solution or the powder [13,14,15]. Spritam^®^—printed using this printing method—shows very rapid oral disintegration because of its highly porous structure and weak bonding between particles. This very rapid disintegration is a new characteristic which arises because 3D printing prepares oral dosage forms without compressing powder. As expected, however, this weak bonding means that the printed medicine tends to show low mechanical strength [13].

One issue with these three types of printing methods is that they require a sufficient solution in the bath or powder in the powder bed to print the object. The final object therefore requires the preparation of an excess amount of solution or powder. We have attempted to minimize the loss of excipient and drug substance by focusing on FDM as a printing method for oral dosage forms.

FDM is a printing method based on fused material extrusion. Thermoplastic polymers in the formation of filaments are used as material. Filament is supplied into the printing nozzle head at a constant speed. It is then heated in the head and the melted materials are extruded from the print head nozzle. The 3D printer extrudes melted material in thin strands and deposits them on a flat plate. The extruded material cools and solidifies soon after deposition, enabling the 3D printer to deposit materials layer by layer along a path determined by the 3D CAD design [16,17]. Because the main ingredients in this process are thermoplastic polymers, some 3D-printed oral dosage forms show sustained release profiles. Among thermoplastic polymers, a poly(vinyl alcohol) (PVA) is widely used as a water-soluble filament [17]. In FDM, the melted thermoplastic polymer is extruded from the heated nozzle and solidifies to form the first layer. From the second layer, the 3D printer can deposit melted material only on the solidified material of the previous layer. In other words, material cannot be deposited in space. If a space is required within the object, PVA-filament is often used to make a basement to support the object. PVA is then deposited to fill the location of the space, and the final object is obtained by dissolving the PVA in water after the printing process [18,19].

There are three reasons why researchers have applied FDM to making oral dosage forms, namely: (1) water solubility, (2) good printability in the field of 3D printing and (3) precedent use of PVA as a pharmaceutical excipient in the pharmaceutical industry [20,21,22]. Compared to powder bed printing, objects printed by FDM tend to show high mechanical strength and an apparent decrease in the risk of cracking and chipping [10]. On the other hand, as an oral dosage form, disintegration is hampered, and rapid drug release is difficult since PVA does not dissolve rapidly [21,23]. To the best of our knowledge, there are only a few FDM printed oral dosage formulations with a rapid drug release profile (more than 85% drug release within 15 min.)

Against this background, our goal has been to prepare fast-dissolving oral dosage forms using FDM 3D printing. We have particularly focused on sugar alcohols, which generally have a good solubility in water and are hardly degraded or color-changed by heat [24]. In particular, sugar alcohols are softened by heat but then harden again on cooling, like thermoplastics. We therefore hypothesized that mixing sugar alcohol with PVA may provide a unique filament which enables the printing of oral dosage forms that have both the rapid drug release properties via FDM with the good printability of PVA.

Here, to test this hypothesis, we prepared dosage forms containing PVA and sugar alcohol by FDM 3D printing and evaluated their in vitro drug release and in vivo bioavailability.

## 2. Materials and Methods

### 2.1. Materials

PVA (Poly(vinyl alcohol), MW 6000, 80% hydrolyzed) was purchased from Polysciences, Inc. (Warrington, PA, USA). Maltitol (SweetPearl P200) and mannitol (Pearlitol 200SD) were purchased from Roquette Japan K.K. (Tokyo, Japan). Xylitol, lactitol (lactitol monohydrate) and sucrose were purchased from FUJIFILM Wako Pure Chemical Corporation (Tokyo, Japan). Erythritol (Erythritol 100M) was purchased from B Food Science Co., Ltd. (Aichi, Japan). Sorbitol was purchased from Kanto Chemical Co., Inc. (Tokyo, Japan). Isomalt (Galen IQ720) was purchased from BENEO-Palatinit GmbH (Mannheim, Germany). Triethyl citrate (TEC) was purchased from Tokyo Chemical Industry Co., Ltd. (Tokyo, Japan). Model drug substance, N_2_-[(2E)-3-(4-chlorophenyl)-2-propenoyl]-N-[2-oxo-2-(4-{[6-(trifluoromethyl) pyrimidine-4-yl]oxy}piperidine-l-yl)ethyl]-3-pyridine-2-yl-L-alaninamide (API, Mw: 617.02, pKa: 4.4, LogD7.4: 4.3, PSA: 78.8, solubility: 5 mg/mL in pH 1.2 buffer solution) and conventional fast dissolving tablet were provided by Astellas Pharma, Inc. (Tokyo, Japan). All materials used in this study were of analytical grade.

### 2.2. Preparation of Filament and Printing Oral Dosage form by FDM

Filaments were prepared by hot melt extrusion. A total of 20 g of drug substance and other excipients were manually mixed in a polyethylene bag. The physical mixture was manually fed into the hopper of a conical twin screw extruder (Xplore Conical Twin Screw Extruder, Model MC15; Xplore, Sittard, The Netherlands) at a barrel temperature of 170 °C with screw rotation at 50 rpm. The extrusion process was continued at a screw speed of 100 rpm in recirculation mode for 3 min each with the extrusion temperature (140–170 °C) adjusted to the formulation component. After the extrusion process, extrudate came out from the die with screw rotation at 10 rpm. The extrudate was stretched and wound using a winding unit, a part of the Micro fibre line: FL (Xplore, Sittard, The Netherlands). Throughout this winding process, extrudate was cooled at room temperature and became filament while the rotation speed of the winding unit was adjusted to achieve a uniform filament diameter.

Oral dosage forms were printed using a commercial FDM 3D printer, Eagleed (Reis Enterprise, Wakayama, Japan), with a nozzle of 0.5 mm diameter. Printing parameters were nozzle travel speed, 50 mm/s; layer height, 0.1 mm; and fill density, 100%. Nozzle temperature was adjusted to the formulation of the filament (140–190 °C).

### 2.3. Rheological Measurement

Viscoelastic properties of the mixture of PVA and sugar alcohol were determined by rheological analysis using a HAAKE Mars 40 rheometer (Thermo Fischer Scientific, Waltham, MA, USA) equipped with parallel-plate geometry with a diameter of 25 mm and a gap of 1 mm. To investigate the complex viscosity (η*), rheological analysis of the mixture was performed in a temperature-dependent manner by a running frequency of 1 Hz with a constant strain of 1%. The rheological data were analyzed using HAAKE Rheowin 4 Job and Data Manager rheological software (Thermo Fisher Scientific, Waltham, MA, USA).

### 2.4. In Vitro Drug Release Test

The drug release test was carried out in accordance with the Japanese Pharmacopeia, Sixteen Edition, using 50 or 100 mg of drug substance in 900 mL of dissolution test 1st fluid (pH 1.2) medium at 37 °C and a paddle speed of 50 rpm. The drug release test was conducted under sink conditions (solubility of the API is 5 mg/mL in pH 1.2 buffer solution). An NTR-6100A dissolution tester (Toyama Sangyo Co., Ltd., Osaka, Japan) was used and drug concentration in the solution was determined with a UV–Vis spectrometer. Absorbance was measured at a wavelength of 285 nm.

### 2.5. In Vivo Oral Absorption Study in Dogs

The animal experiments in this study were approved by the Institutional Animal Care and Use Committee of Astellas Pharma, Inc., and were performed in accordance with animal welfare guidelines.

Sample was orally administered to beagle dogs, followed immediately after by up to 50 mL of water by a catheter. Then, 2.5 mL of blood was collected at each of 8 time points, namely 0.25, 0.5, 1, 2, 4, 6, 8 and 24 h post oral administration. Blood plasma was obtained by centrifugal separation at 1870× g for 15 min. Four dogs (*n* = 4) were used for each sample. The dogs were fasted from 16 h before administration until the completion of blood collection at 8 h after administration. Further, the dogs were restricted from drinking water from 30 min before administration until the completion of blood collection at 2 h after administration. To adjust pH in the stomach, intramuscular administration of pentagastrin (0.015 mg/kg) was performed 30 min before and 30 and 90 min after administration [25].

In vivo plasma samples were extracted using a simple protein precipitation method by adding diethyl ether (4 mL) and pH10 carbonate-bicarbonate buffer to samples (0.5 mL). Benzophenone was used as an internal standard (100 µg/mL in methanol). Samples were mixed by shaking for 20 min and centrifuged at 1630× g for 20 min. The supernatant organic phase was collected and evaporated. After evaporation, it was re-dissolved in 500 µL of mobile phase (acetonitrile/0.02 M KH_2_PO_4_ (55/45)). Then, 20 µL of this solution was injected and analyzed using HPLC (e2695, Waters Co., MA, USA with an ODS column (Inertsil ODS-3, 5 µm, 150 × 4.6 mm I.D., GL Sciences, Tokyo, Japan). The limit of detection was 0.2 µg/mL and the assay range used was 0.2–20 µg/mL.

Pharmacokinetic parameters were computed by the non-compartmental model using Phoenix WinNonlin (Certara, Pharsight Corporation, Moutain View, CA, USA).

### 2.6. Statistical Analysis

The results are expressed as the mean ± standard deviation (SD) (*n* = 3) or mean ± range (*n* = 2). Paired *t*-test was used to compare pharmacokinetic parameters between 3D-printed oral dosage form and conventional tablet in the dog study. Differences were considered significant at *p* < 0.05.

## 3. Results and Discussion

### 3.1. Preparation of a 3D-printed Oral Dosage Form Containing Maltitol

As the aim of this study was to prepare a 3D-printed rapidly dissolving medicine, we selected a low molecular weight PVA as the main base material because of its water-soluble feature. We attempted to increase the drug release rate by adding sugar alcohol to the PVA-based filament. We selected maltitol as sugar alcohol to verify the possibility of (1) making filament with a mixture of sugar alcohol and PVA, and (2) improving the drug release rate from the printed dosage form with the filament.

#### 3.1.1. Preparation of Filament Contains Maltitol and Printing Oral Dosage Form

**Figure 1:**
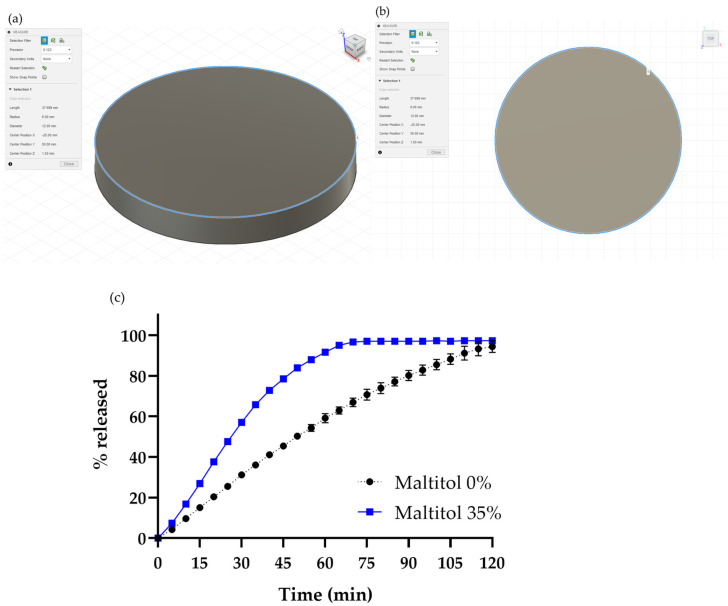
3D CAD design of a cylindrical shape (12 mm diameter and 1.5 mm height), (**a**) perspective view and (**b**) top view. (**c**) Drug release profiles of the oral dosage form printed using PVA-based filament not containing maltitol (●) and filament containing maltitol(■). Data represent mean ± range, n = 2.

A physical mixture containing 35% maltitol (Table 1) was manually prepared. API was selected as the model drug substance to evaluate the drug release profile from printed oral dosage form. Filament was successfully prepared through the extrusion process with the conical twin screw extruder and winding process while adjusting winding speed. The filament was set to the FDM printer and used to print a cylindrically shaped object with a diameter of 12 mm based on 3D CAD data (Figure 1a). The height of the object was 1.5 mm. The oral dosage form was printed successfully with PVA-filament both with and without maltitol. The printing temperatures were 165 °C for PVA-filament with maltitol and 190 °C for the filament without maltitol. These results showed that sugar alcohol could be added to PVA without losing the formability of the PVA.

#### 3.1.2. Drug Release Study of the Maltitol-PVA Oral Dosage Form

The drug release profiles of the oral dosage printed by PVA-filament and maltitol-PVA-filament are shown in Figure 1b. Although the drug release rate was improved by adding maltitol, as intended, it still did not meet the fast-dissolving profile of our target release rate (more than 85% drug release within 15 min). Observation of the drug release behavior revealed that the dosage form did not disintegrate and showed the behavior of a fully dissolving property. As the surface area of an object can influence its drug release rate [19,20,23], we thought it would be useful to change the shape of the object.

#### 3.1.3. Changing Shape of the Oral Dosage Form

**Figure 2:**
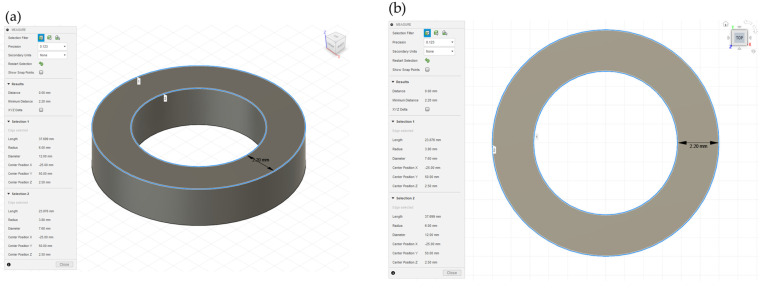

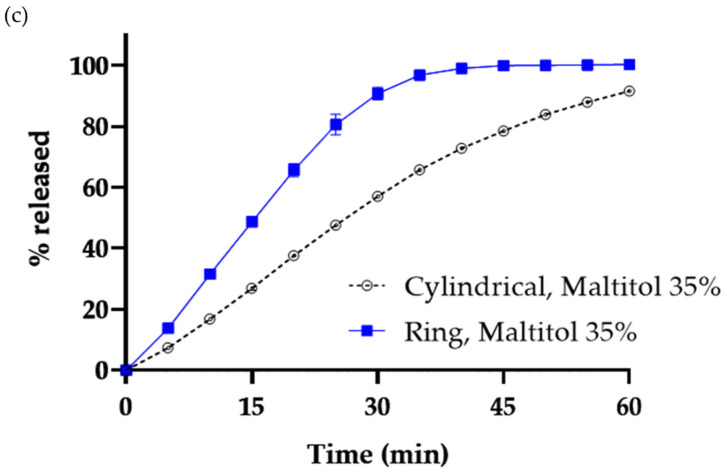
3D CAD design of ring shape (diameter of the outer circle is 12 mm, diameter of the inner circle is 7.6 mm, and 2.5 mm height), (**a**) perspective view and (**b**) top view. (**c**) Drug release profiles of the 3D-printed oral dosage form in the cylindrical shape (●) and ring shape (■). Data of the cylindrical shape represent the mean ± range, *n* = 2; data of the ring shape represent the mean ± SD, *n* = 3.

One advantage of 3D printing is that it is easy to change the shape of objects based on 3D CAD design. We changed the model shape from a cylindrical shape to a ring shape (Figure 2a). Ring-shaped objects were also printed successfully using maltitol-PVA filament. The drug release profiles of the cylindrical and ring shapes are shown in Figure 2b.

The ring-shaped object showed a faster drug release rate than the cylindrical object. Note that when the volumes of the cylindrical shape (Figure 1a), with a height of 1.5 mm, and the ring shape (Figure 2b) are held constant, the height of the ring shape is 2.5 mm, giving, respective, theoretical surface areas of 283 mm^2^ and 289 mm^2^. This means there was no actual increase in the surface area of the ring-shaped object compared with the cylindrical shape, but rather that the drug release rate was improved. In the previous reports, the drug release rate depended on the surface area/volume ratio and the surface area of the tablets [23,26]. We considered that the bottom surface of each object contributes to drug release in only a limited way in this study, because the objects stayed at the bottom of the vessel and did not move during the drug release test. The available surface area excluding the bottom areas of the cylindrical and ring objects are 170 mm^2^ and 222 mm^2^, respectively. The ring-shaped object showing a faster drug release profile than that of the cylindrical shape is considered to be the result of increasing the available surface area of printed object.

Additionally, our findings suggested that the drug release rate of the oral dosage form could be changed by changing its shape, even without changing its formulation composition. This would be an additional value of using 3D printing technology for the manufacture of oral dosage forms.

### 3.2. Screening of Sugar Alcohol

We confirmed that we could prepare a PVA filament containing maltitol and that this could improve the drug release speed of an oral dosage form printed by the filament. Nevertheless, we did not obtain a rapid drug release object which met our criteria. We therefore screened other sugar alcohols to find a more effective one [27], and prepared filament containing the sugar alcohols, xylitol, mannitol, lactitol, erythritol, sorbitol and isomalt (Composition: 20% API, 40% PVA, 35% sugar alcohol and 5% TEC). We also used sucrose, which is not a sugar alcohol and has high solubility of 2.1 g/mL at 20 °C as a reference.

**Figure 3:**
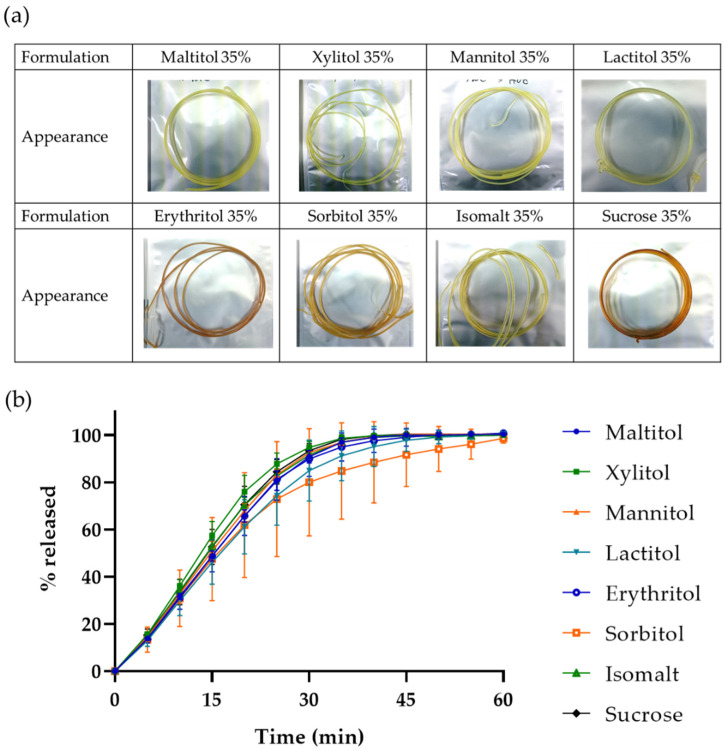
(**a**) Appearance of filament containing each sugar alcohol. (**b**) Drug release profiles of 3D-printed oral dosage forms in a ring shape, which printed with filament containing the respective sugar alcohol. Data represent mean ± SD, *n* = 3.

The results showed that filament could be prepared using any sugar alcohol and sucrose. The appearance of these filaments is shown in Figure 3a. The filament diameters were comparable, in the range of 1.57 to 1.83 mm, and the RSD value was less than 9% for any sugar alcohol used (average of 10 measurements). These filaments could therefore be used for 3D printing. The addition ratio of sugar alcohol to PVA is approximately 1:1, and we confirmed that any of these sugar alcohols could be added to the PVA filament, and these sugar alcohol-PVA-filaments could be used in FDM without problems if this addition ratio and manufacturing method were used.

We then used these filaments to print ring-shaped objects (Figure 2a), and found that all the filaments could do so while adjusting the printing temperatures to each formulation (maltitol, 165 °C; xylitol, 150 °C; mannitol, 140 °C; lactitol, 155 °C; erythritol, 140 °C; sorbitol, 155 °C; isomalt, 150 °C; sucrose, 180 °C). The drug release profiles are shown in Figure 3b. No significant difference in the drug release rate was observed between any sugar alcohol, although the sorbitol-based object showed a slightly slower drug release. Sorbitol is known as one of the most hygroscopic sugar alcohols [28], and given that the drug release rate of the sorbitol-based object showed a slight variation compared with the other sugar alcohols, moisture absorption by the sorbitol-based printed object might have affected its drug release profile.

### 3.3. Rheological Measurement of the Sugar Alcohol and PVA Mixture

In this study, we used a conical twin screw extruder for the extrusion process. The extruder enables the melt kneading process in a closed system. The equipment is suitable for small-scale applications such as formulation screening and the closed system makes it relatively easy to obtain well-mixed extrudates [29]. Twin screw extruders are often used in pharmaceutical manufacturing under a continuous process. Typically, they consist of a barrel enclosing two screws. The powder mixture is fed into a hopper, and is then transported with kneading along the barrel by the rotation of the screws [30], exiting from the other end as a kneaded and extruded material. This process is performed continuously. Since the materials gradually melt during transportation along the barrel, mixing defects may occur if the melting points of the components significantly differ [30,31]. In addition, the melting/kneading process is hampered if the melt viscosity of the materials is extremely low or high. Kolter et al. mentioned that the complex viscosity of the melted material should be in the range of 1000–10,000 Pa·s in the extrusion process [32]. Because we found that none of the sugar alcohols was strongly effective in improving the drug release speed of the PVA-based and -printed oral dosage forms (Figure 3b), we continued to use maltitol, and measured the complex viscosity of the mixture of maltitol and PVA to confirm whether maltitol is potentially suitable for future use.

**Figure 4:**
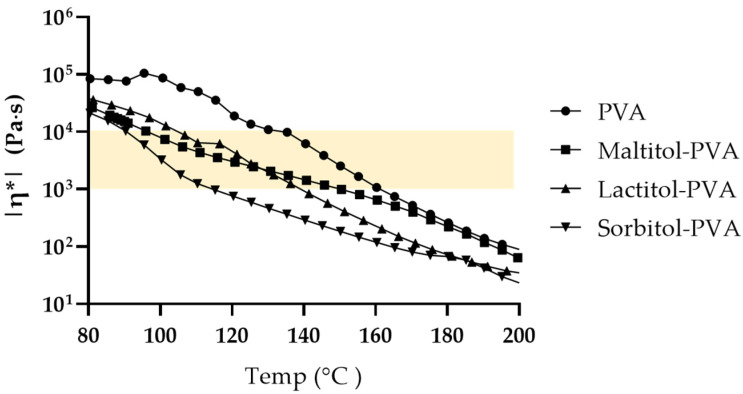
Complex viscosity (|η*|) data of each powder mixture; PVA only (●), maltitol/PVA/TEC = 35/60/5 (■), lactitol/PVA/TEC = 35/60/5 (▲) and sorbitol/PVA/TEC = 35/60/5 (▼).

Physical mixtures of sugar alcohol, PVA and TEC were prepared manually in a 35:60:5 ratio. In addition to maltitol, lactitol and sorbitol were used as comparative controls. After melting at 200 °C, the viscoelasticity of the mixture was measured using a rheometer while the temperature was lowered. The results are shown in Figure 4. Sorbitol-PVA showed the lowest level of complex viscosity among samples at any temperature in the range measured. Lactitol (Tm: 150 °C) and maltitol (Tm: 148 °C) melt at about the same temperature, but the complex viscosity of lactitol-PVA was lower than that of maltitol-PVA over 140 °C. In addition, the complex viscosity of maltitol-PVA remained in the preferred extrusion range when the temperature was between 95 to 150 °C. In contrast, sorbitol and lactitol maintained the preferable range when the temperature was between 90 to 115 °C and between 100 and 135 °C, respectively. Given that the physical mixture melts gradually in the barrel of the extruder and that the process temperature depends on the material having the highest melting point among the formulation components, it is preferred that the complex viscosity of the melted material is in a suitable range for extrusion, even if this temperature is high. These considerations indicated that maltitol was suitable for use as a sugar alcohol.

### 3.4. Effect of the Addition Ratio of Maltitol on Filament Formulation

As described above, a filament which contains 35% maltitol (other composition: 20% API, 40% PVA and 5% TEC) could be made, and we confirmed that the drug release speed of the printed oral dosage form increased with the addition of maltitol. To further increase the drug release speed, it was considered reasonable to increase the addition ratio of maltitol since it has a higher water solubility than PVA. A filament containing 55% maltitol (other composition: 20% API, 20% PVA and 5% TEC) was successfully prepared, but preparation was difficult when the maltitol ratio was increased to 65% (other composition: 20% API, 10% PVA and 5% TEC). More specifically, extrudate which contained 65% maltitol showed a slow solidification speed after exiting from the die of the extruder, and tension could not be applied in the winding process. A possible reason for this is that it takes a long time to solidify the extrudate with a decreasing temperature when the amount of PVA added is low. To avoid this, the addition of a certain amount of PVA is necessary to prepare the filament, and in this case, this was 20%.

Finally, we prepared three filaments with different addition ratios of maltitol (20%, 35% and 55%). We were able to print an oral dosage form in the shape of a ring (Figure 2a) successfully with each filament at a printing temperature of 165 °C. As shown in Table 2, the weight of the printed object increased as the amount of added maltitol increased, despite the use of the same 3D CAD design. This might be due to the change in density of the filament caused by the addition of maltitol.

**Figure 5:**
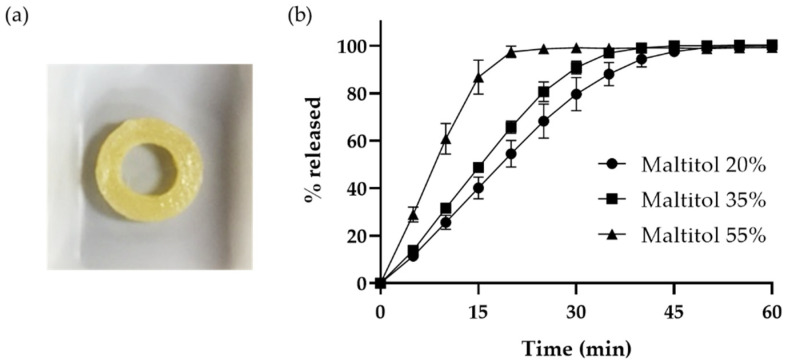
(**a**) Appearance of 3D-printed oral dosage form in a ring shape using a filament containing 55% of maltitol. (**b**) Drug release profiles of 3D-printed oral dosage form in a ring shape using a filament containing 20% (●), 35% (■) and 55% (▲) of maltitol. Data represent mean ± SD, *n* = 3.

The drug release profiles of these dosage forms are shown in Figure 5. As expected, the drug release speed increased as the rate of the addition of maltitol increased. In contrast, the printed oral dosage form containing 55% maltitol showed a greater than 85% drug release in 15 min (maltitol 20%: 40.1% drug released, maltitol 35%: 48.7% drug released, maltitol 55%: 86.8% drug released in 15 min). This result met our target drug release rate, and we were able to prepare a 3D-printed oral dosage form that showed drug release rates comparable to those of conventional tablets generally considered to be fast-dissolving tablets.

### 3.5. In Vivo Oral Absorption Study in Dogs

**Figure 6:**
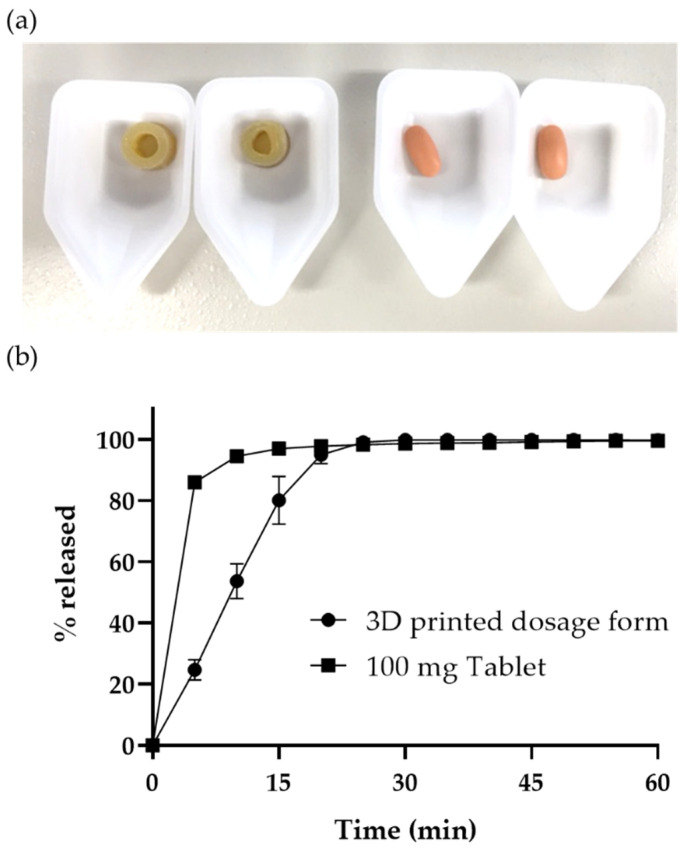
(**a**) Appearance of the 3D-printed oral dosage forms (**left**) and conventional tablets (**right**). (**b**) Drug release profiles of the 100 mg 3D-printed oral dosage form (●) and conventional tablet (■). Data represent mean ± range, *n* = 2.

The oral dosage form printed by FDM using a filament which contained 55% maltitol and 20% PVA showed a fast-dissolving profile. However, this form’s drug release behavior was the dissolving type, and not the disintegrating type like conventional fast-dissolving tablets. To further promote the application of FDM for manufacturing medicines it was important to verify whether this printed object would release a drug as fast in vivo as in vitro. The model API used in this study was a drug substance which was developed as a 100 mg tablet, which had a fast drug release. To verify the oral absorption of the printed oral dosage form, we prepared 100 mg of the printed oral dosage form using a filament which contained 55% maltitol (20% API, 20% PVA and 5% TEC). To adjust the drug substance amount in the printed oral dosage form to 100 mg, we printed it in the shape of a ring (Figure 2b) of 5.2 mm height. The drug release profiles of the 3D-printed oral dosage form and the 100 mg conventional tablet are shown in Figure 6b. The conventional tablet showed a fast-dissolving profile, while the 3D-printed oral dosage form showed a drug release of 80% in 15 min. To prepare 100 mg printed dosage forms, we doubled the volume by doubling their height compared with the printed dosage forms, which showed 85% drug release in 15 min (Figure 5). However, the surface area increased only 1.5 times when the height (volume) was doubled, and the available surface area increased 1.7 times. It means that the value of available surface area/volume became 0.85 times larger when the height was doubled. This might lead to a decrease in the drug release rate from the 100 mg 3D-printed oral dosage forms.

**Figure 7:**
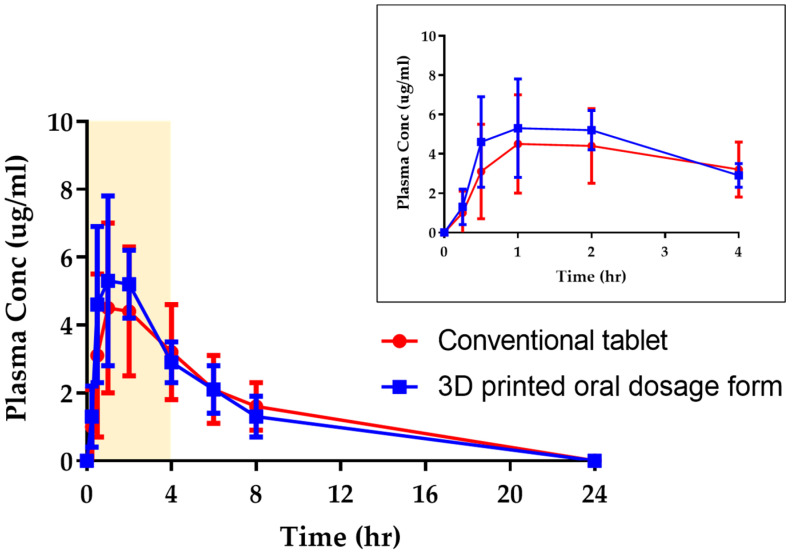
Plasma drug concentrations after oral administration of the conventional tablet (●) and 3D-printed oral dosage form (■) to beagle dogs. Data represent mean ± SD, *n* = 4.

The oral absorption of these medicines was evaluated in beagle dogs (*n* = 4). The mean plasma concentrations of drug substances are shown in Figure 7, and *C*_max_, *T*_max_ and AUC_0–24 h_ of each medicine in Table 3. There were no significant differences in pharmacokinetic parameters between the 3D-printed oral dosage form and the conventional tablet. The 3D-printed oral dosage form showed comparable AUC_0–24 h_ and *T*_max_ to the conventional tablet. It also showed a slightly higher *C*_max_, but the difference was not statistically significant. This means that the 3D-printed oral dosage form composed of maltitol and PVA dissolves rapidly in vivo as well as in vitro.

## 4. Conclusions

This study showed that a sugar alcohol could be added to a PVA-based filament and the sugar alcohol-PVA-filament could be used for FDM. These filaments enabled an increase in the drug release rate from an oral dosage form printed using the filament. It was confirmed that maltitol is suitable as a sugar alcohol in terms of the complex viscosity and the drug release rate from printed oral dosage forms was improved by increasing the amount of maltitol added and changing the shape of object. A 3D-printed oral dosage form containing 55% maltitol in a ring shape showed a more than 85% drug release in 15 min in vitro. Further, this oral dosage form showed a similar level of oral absorption in vivo to a conventional fast-dissolving tablet.

This study also showed that the drug release profile of an oral dosage form printed with a maltitol-PVA-based filament could be controlled by changing the available surface area of objects, even if they had the same formulation composition. Changing the shape of a medicine also leads to a change in the amount of drug it contains. Controlling the drug release profile and changing the drug amount of an oral dosage form by manufacturing parameters (changing 3D CAD design) only will contribute to the promotion of personalized medicine. Three-dimensional printing technology which enables the printing of fast-dissolving oral dosage forms will have a major impact on the pharmaceutical industry.

## Figures and Tables

**Table 1:** Composition of the formulation of PVA-filament with or without maltitol. Thickness and weight of the printed oral dosage form. Data of thickness and weight represent means ± range, *n* = 2.

Formulation	Maltitol 0%	Maltitol 35%
API	20	20
PVA	79	40
Maltitol	-	35
TEC	1	5
Thickness (mm)	1.59 ± 0.01	1.56 ± 0.11
Weight (mg)	208.0 ± 0.5	239.2 ± 12.9

**Table 2:** Thickness and weight of the printed oral dosage form printed from maltitol-PVA filament with various addition ratio. Date represent mean ± SD, *n* = 3.

Formulation	Maltitol 20%	Maltitol 35%	Maltitol 55%
Thickness (mm)	2.36 ± 0.09	2.62 ± 0.18	2.59 ± 0.13
Weight (mg)	229.5 ± 2.4	245.6 ± 6.8	263.2 ± 30.3

**Table 3:** Pharmacokinetic parameters in the oral absorption study in dogs.

Samples	*C*_max_ (µg/mL)	*T*_max_ (h)	AUC_0–24 h_ (µg·h/mL)
Conventional tablet	4.2 ± 2.2	1.4 ± 0.6	34.0 ± 11.1
3D-printed oral dosage form	5.6 ± 1.8	1.2 ± 0.8	35.1 ± 15.6

## Data Availability

The data presented in this study are available from the corresponding author upon reasonable request.
